# Factors associated with blooms of cyanobacteria in a large shallow lake, China

**DOI:** 10.1186/s12302-018-0152-2

**Published:** 2018-07-28

**Authors:** Di Li, Naicheng Wu, Song Tang, Guanyong Su, Xuwen Li, Yong Zhang, Guoxiang Wang, Junyi Zhang, Hongling Liu, Markus Hecker, John P. Giesy, Hongxia Yu

**Affiliations:** 1 State Key Laboratory of Pollution Control and Resource Reuse, School of the Environment, Nanjing University, Nanjing, Jiangsu 210046 China; 2 Jiangsu Environmental Monitoring Center, Nanjing, Jiangsu 210036 China; 3 Department of Hydrology and Water Resources Management, Kiel University, Kiel, 24118 Germany; 4 National Institute of Environmental Health, Chinese Center for Disease Control and Prevention, Beijing, 100021 China; 5 Jiangsu Key Laboratory of Chemical Pollution Control and Resources Reuse, School of Environmental and Biological Engineering, Nanjing University of Science and Technology, Nanjing, 210094 China; 6 School of the Environment, Nanjing Normal University, Nanjing, Jiangsu 210023 China; 7 Wuxi Environmental Monitoring Center, Wuxi, Jiangsu 214000 China; 8 Toxicology Centre, University of Saskatchewan, Saskatoon, SK S7N 5B3 Canada; 9 School of Environment and Sustainability, University of Saskatchewan, Saskatoon, SK S7N 5C3 Canada; 10 Department of Veterinary Biomedical Sciences and Toxicology Centre, University of Saskatchewan, Saskatoon, SK S7N5B3 Canada; 11 Department of Zoology and Center for Integrative Toxicology, Michigan State University, East Lansing, MI 48824 USA; 12 School of Biological Sciences, University of Hong Kong, Hong Kong, SAR China

**Keywords:** Eutrophication, Nutrients, Phosphorus, Nitrogen, Phytoplankton productivity, Diversity, Microcystin-LR

## Abstract

**Background:**

Eutrophication of freshwater systems can result in blooms of phytoplankton, in many cases cyanobacteria. This can lead to shifts in structure and functions of phytoplankton communities adversely affecting the quality of drinking water sources, which in turn impairs public health. Relationships between structures of phytoplankton communities and concentrations of the toxicant, microcystin–leucine–arginine (MC-LR), have not been well examined in large shallow lakes. The present study investigated phytoplankton communities at seven locations from January to December of 2015 in Tai Lake, and relationships between structures and diversities of phytoplankton communities and water quality parameters, including concentrations of MC-LR and metals, were analyzed.

**Results:**

A total of 124 taxa of phytoplankton were observed, and the predominant taxa were *Microcystis* sp. and *Dolichospermum flos*-*aquae* of *Cyanophyta* and *Planctonema* sp. of *Chlorophyta*. The greatest diversities of phytoplankton communities, as indicated by species richness, Simpson, Shannon–Wiener, the Berger and Parker, and the Pielou evenness indices, were observed in spring. Furthermore, productivity of phytoplankton was significantly and negatively correlated with diversities. These results demonstrated that Simpson, Shannon–Wiener, the Berger and Parker, and the Pielou evenness indices of phytoplankton communities were significantly related to trophic status and overall primary productivity in Tai Lake. In addition, temperature of surface water, pH, permanganate index, biochemical oxygen demand, total phosphorus, arsenic, total nitrogen/total phosphorous ratio, and MC-LR were the main factors associated with structures of phytoplankton communities in Tai Lake.

**Conclusion:**

The present study provided helpful information on phytoplankton community structure and diversity in Tai Lake from January to December of 2015. Our findings demonstrated that Simpson, Shannon–Wiener, the Berger and Parker, and the Pielou evenness indices could be used to assess and monitor for status and trends in water quality of Tai Lake. In addition, MC-LR was one of the main factors associated with structures of phytoplankton communities in Tai Lake. The findings may help to address important ecological questions about the impact of a changing environment on biodiversity of lake ecosystems and the control of algae bloom. Further studies are needed to explore the relationship between MC-LR and phytoplankton communities in the laboratory.

**Electronic supplementary material:**

The online version of this article (10.1186/s12302-018-0152-2) contains supplementary material, which is available to authorized users.

## Background

Biodiversity represents the complexity of life and includes phenotypic, genotypic, taxonomic and ecological diversity [1]. Characterization of biodiversity of phytoplankton communities can help managers and researchers understand the status and trends in changes in the structure of ecosystems, e.g., due to stressors such as contamination with pollutants and nutrients [2]. Therefore, understanding the ecological processes, as well as abiotic and biotic factors that contribute to absolute and relative abundances of taxa in these communities are major goals of basic and applied community ecology. Metrics, such as species richness and the Shannon–Wiener diversity index, represent important tools for the characterization of changes in phytoplankton communities in aquatic ecosystems [3–5].

Compositions of phytoplankton communities are crucial determinants of structures of food webs in aquatic ecosystems, and because of their rapid responses to environmental stressors such as pollution these are considered to be important environmental indicators [6–9]. Results of previous studies have suggested that water temperature (WT), water level, seasonality, optical properties, and nutrients, especially nitrogen (N) and phosphorus (P) are the main factors that affect the composition of phytoplankton communities [9–15]. Hydrodynamic force, mixing depth, and euphotic depth were also important physical factors that influenced phytoplankton dynamics and bloom condition [16, 17]. Species compositions and richness of phytoplankton can also be altered by exposure to pollution, such as metals [18, 19]. Thus, to protect biodiversity and restore aquatic ecosystems, there is a need for regular monitoring to ascertain that these water quality parameters are within acceptable levels, and it is important to understand the impact of the changing environment on the structure of phytoplankton communities. Although productivity of phytoplankton, expressed as concentrations of Chlorophyll *a*, has been suggested as a robust indicator for assessment of quality of ecosystems [20], relationships between productivity and composition and diversity of phytoplankton have been seldom studied in aquatic ecosystems.

Due to human activities and climate changes, eutrophication of lakes have been global problems, and can cause significant shifts in phytoplankton communities resulting over-dominance of unwanted taxa, such as cyanobacteria [21]. Cyanobacteria blooms are expected to become more frequent, of greater severity and duration worldwide [22], posing serious threats to the health of many surface water ecosystems, and as such affecting the safety of drinking water sources [23]. Because of their unique characteristics, such as adapting low or high light, in the presence of excess nutrients, cyanobacteria have competitive advantages of many other species of plankton. Several cyanobacteria, such as *Microcystis* spp., *Dolichospermum flos*-*aquae*, and *Oscillatoria tenuis*, can produce toxins [24, 25], such as microcystins (MCs) [26], microcystins, especially microcystin-LR (MC-LR; L for leucine and R for arginine), are widely distributed across eutrophic freshwater ecosystems and have been shown to be toxic to a wide range of aquatic organisms and humans [27–31]. However, the impacts of MC-LR on structure and diversity of aquatic communities specifically phytoplankton communities have been rarely reported in natural waters [5, 32].

As the third-largest lake in China, Tai Lake is a shallow lake that is a critical source of drinking water for several populous cities of China. However, due to numerous anthropogenic stressors, between 1998 and 2007, a number of blooms of cyanobacteria occurred throughout the year, except for January and February [33]. Between 2008 and 2011, the frequency of algae blooms was increasing, and blooms occurred even in January [34]. Blooms of cyanobacteria first appeared in Meiliang Bay of Tai Lake in the 1980s [35], and previous studies found that absolute and relative density of phytoplankton in Tai lake was highest in this bay [36–38]. However, phytoplankton in the other areas of Tai Lake was seldom surveyed. In addition, since the most common freshwater cyanobacterium, *Microcystis* spp. [39], is also a dominating and problematic species in Tai Lake, this species has been extensively researched [40–42]. However, effects of the dominant taxa on the other taxa of phytoplankton during cyanobacteria blooms were seldom studied in the lake.

In the present study, variations in environmental conditions and communities of phytoplankton were investigated monthly at seven different locations of Tai Lake in 2015. The aims of this study were to (1) explore potential effects of environmental parameters on the structures of phytoplankton communities; (2) determine relationships between phytoplankton productivity and diversity: and (3) identify factors driving changes in dominant taxa of phytoplankton. Addressing these important ecological questions could help managers better understand biodiversity of plankton community and better make decision to protect this important shallow lake.

## Methods

### Study area

**Fig. 1:**
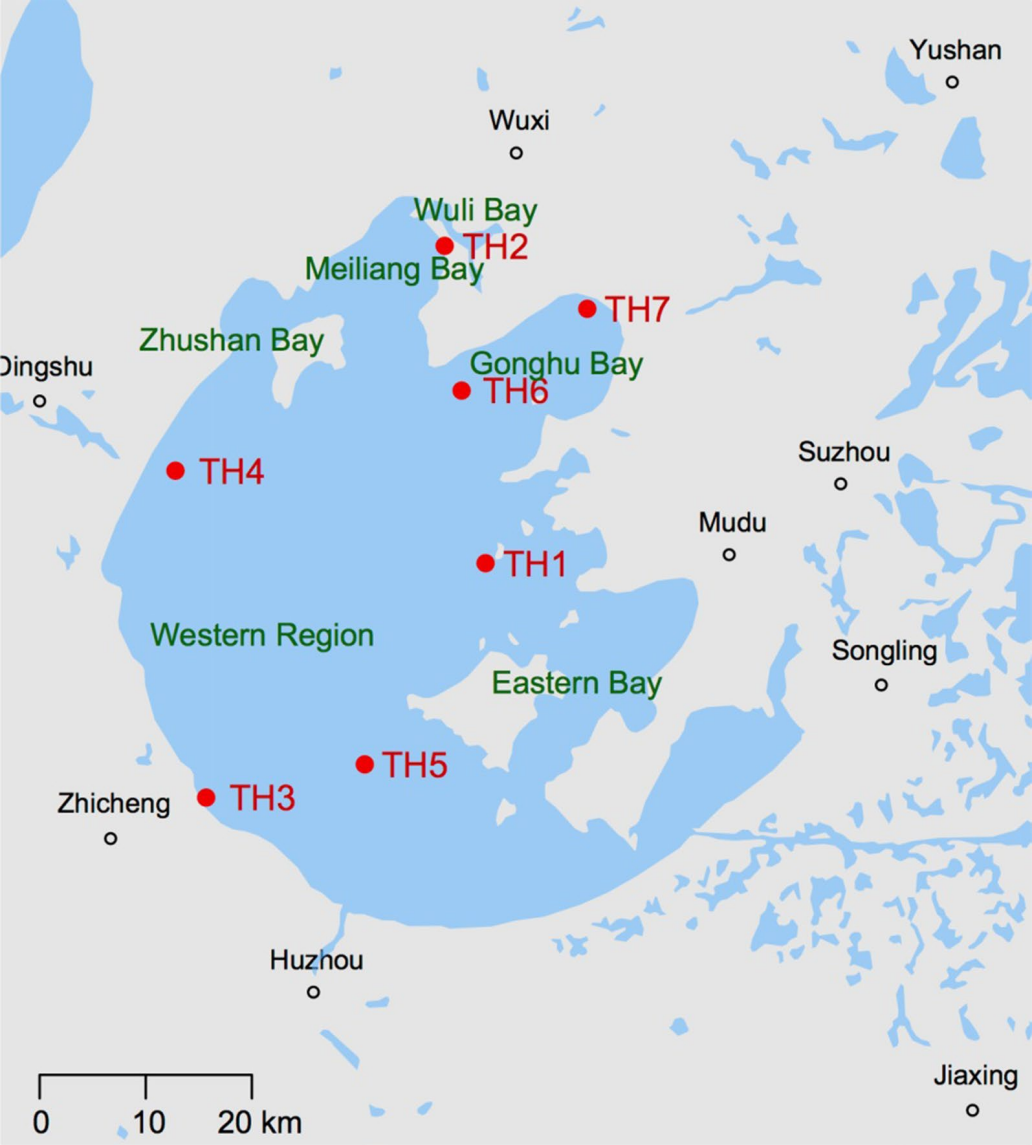
Locations of seven sampling sites in Tai Lake

Tai Lake is located between Jiangsu and Zhejiang Province. Communities of phytoplankton were investigated monthly at seven different sites, which are two of the most densely populated regions in China. Tai Lake has an area of 2338 km^2^, with a maximum length and width of 68.5 and 56 km, respectively. The average depth is approximately 1.9 m and the average annual air temperature is between 16.0 and 18.0 °C. The annual mean precipitation is between 1100 and 1150 mm. In the present study, seven sampling locations were established in Tai Lake (Fig. 1), which represented areas of differing ecological characteristics across the entire Lake. Specifically, they were in the eastern bay (TH1), Meiliang Bay (TH2), southwest area of the lake (TH3), northwest area of the lake (TH4), central lake (TH5), and Gonghu Bay (TH6 and TH7).

### Sampling

#### Collection of water and analyses

Sampling was performed at seven locations each month from January to December in 2015. In brief, water was collected from a depth of 0.5 m below the surface, stored in glass containers at 0–4 °C in the dark, brought to the laboratory, and processed within 12 h for each sample based on standard methods [43]. Water temperature (WT), pH, dissolved oxygen (DO), and conductivity were measured in situ by use of YSI water quality sondes (YSI Incorporated, 6600V2-4, Ohio, USA). Water transparency (SD) was determined with a 30-cm Secchi disk. Analysis of COD_Cr_, COD_Mn_, TP, TN, BOD_5_, NH_4_^+^-N, F^−^, As, Pb, Cu, and Chl *a* in water samples was based on a standard method [43]. Enzyme-linked immune sorbent assay (ELISA) was used to measure concentrations of MC-LR [44, 45], and the assay kits were bought from institute of hydrobiology of Chinese academy of sciences. The TN/TP ratio (NPR) was calculated. Based on five limnological parameters, including SD, COD_Mn_, TP, TN, and Chl *a*, the synthesized trophic state index (STSI) was calculated for each water sample and used to assess eutrophication status (Additional file 1: Table S1) [46–48].

#### Collection of phytoplankton and analyses

In brief, 1000 mL samples of water were collected at each location, and phytoplankton was fixed in acid Lugol’s solution and transported to laboratory at 4 °C [43]. Identification of phytoplankton was performed to the species level and enumerated by counting at least 30 random fields in Sedgewick-Rafter sedimentation chambers (30 mL) using an inverted microscope (BX53, Olympus, Japan) [43, 49–51]. Numbers of cells of each taxon, as well as dimensions of individuals, including maximum linear dimension, were estimated. The dominant taxa were identified in the following equation [52]:

1 $$ Y = \, \left( {n_{i} /N} \right)f_{i} , $$

where *n*_*i*_ is the number of cells of the *i*th taxa, *f*_*i*_ is the frequency of the *i*th taxa appearing at the survey sites; *N* is the number of phytoplankton taxa observed during the study. When *Y* > 0.02, the taxa were classified as being dominant in the phytoplankton community.To determine diversity of phytoplankton communities, commonly used diversity indicators, included species richness (*S*), the Shannon–Wiener index (*H*′), the Simpson index (*D*_s_), the Berger and Parker index (*D*_b_), and Pielou evenness index (*J*) were calculated by PRIMER software version 6.1.10 (Lutton, Ivybridge, United Kingdom).

Species diversity was estimated by Shannon–Wiener index, Simpson index, and Berger–Parker index, which are members of Renyi diversity family, as given the following equation [53, 54]:

2 $$ {\text{HR}}_{\alpha } = \frac{1}{1 - \alpha }{ \log }\mathop \sum \limits_{i = 1}^{s} Pi^{\alpha } . $$

This is a one-parametric diversity index family in which diversity of an assemblage is characterized by a diversity profile instead of a numerical value (Additional file 1: Fig. S1). By increasing the scale parameter (*α*), contributions of abundant species to the diversity of the assemblage increase, while contributions of rare species decrease. Researchers who want the index to be sensitive to the compositions of more abundant species but relatively indifferent to that of more rare species can use the diversity indices of Renyi family [55]. The values of Renyi diversity are lgS, *H*′, *D*_s_, and *D*_b_ when the following *α* values were used 0, 1, 2, and ∞ respectively.

### Data analyses

Differences in water quality parameters or phytoplankton indicators during months or four seasons, and among seven sites in Tai Lake were analyzed by use of the Kruskal–Wallis, non-parametric test (SPSS 22.0, Chicago, Illinois, USA). A linear regression analysis was used to elucidate relationships between phytoplankton productivity (Chl *a*) and diversity metrics in Tai Lake (SPSS 22.0, Chicago, Illinois, USA). A *p* value < 0.05 was used as the threshold for statistical significance. The Multi-Response Permutation Procedure (MRPP, *mrpp* function in R package *vegan*) was used to determine differences in community composition and structure among locations and months. The null hypothesis was that there was no difference among the groups in a Monte Carlo randomization procedure with 999 permutations. To explore potential effects of environmental parameters on phytoplankton communities (*Objective 2*), the following preliminary data analyses were conducted. First, rare species with relative abundance < 0.5% when all samples were summed were excluded; this requirement reduced the number of taxa in the analysis from 124 to 31. Second, species counts were converted to relative abundances (0–100%), which were Hellinger transformed to reduce the weight of abundant species, while preserving Euclidean distances between samples in the multidimensional space [56]. Third, environmental variables with significant multicollinearity (with variance inflation factor > 10 and Spearman’s rank correlation coefficient |*r*| ≥ 0.75) were excluded. STSI was removed due to its greater correlations with TP (Additional file 1: Table S2) and the other 17 environmental variables were included in the following analyses. A preliminary detrended correspondence analysis (DCA) on the species data produced a longest gradient length of 3.18 along the first axis, which suggested that redundancy analysis (RDA) was appropriate [57]. RDA was performed by use of the *rda* function and tested for significance using the *anova* function. Only when it was significant, forward selection and Monte Carlo permutations (999 iterations) were used to select variables that significantly (*p *< 0.05) explained the variance in species composition. Forward selection was performed by use of the *envfit* function in R package *vegan*. Furthermore, generalized linear models (GLMs) with Gaussian error distribution [58] were used to examine influencing factors of different algal indices (e.g., diversity indices, the dominant taxa) (*Objective 3*). The best approximating model was selected, based on Akaike’s information criterion (AIC) [59], using function *stepAIC* in R package *MASS*. GLMs were performed with all environmental variables, except for STSI which was excluded due to its greater correlations with TP (see also above). The reason GLMs were selected rather than univariate analyses was because GLM models interpret importance of variables in a multivariate setting [60]. All these analyses were performed with R software version 3.3.2.

## Results

### Water quality parameters

Mean values of water quality parameters varied among seven sampling locations (Table 1). Concentrations of Chl *a*, measuring of phytoplankton productivity that covered the whole trophic spectrum, ranged between 5.0 and 362 μg/L in Tai Lake. The largest synthesized trophic state index (STSI) was observed at TH4, while the least was observed at TH1. Greatest concentrations of MC-LR (0.352 μg/L) were observed at TH2, and least concentrations (0.069 μg/L) were observed at TH1. For concentrations of pollutants at the seven locations, TH4 seemed to be most contaminated, whereas TH1 (the eastern region) and TH5 (the central lake) were less contaminated (*p *< 0.000001).

**Table 1:** Monthly values (mean ± SD) of water parameters at seven sampling sites from January to December of 2015 in Tai Lake

Items	TH1	TH2	TH3	TH4	TH5	TH6	TH7	*P*
WT (°C)	17.6 ± 8.7	18.8 ± 9.0	17.3 ± 9.1	17.6 ± 8.8	17.1 ± 8.4	18.6 ± 8.9	18.7 ± 8.3	0.999
pH	8.22 ± 0.34	8.49 ± 0.42	8.16 ± 0.46	8.11 ± 0.42	8.18 ± 0.44	8.48 ± 0.33	8.29 ± 0.29	0.113
SD (m)	0.33 ± 0.05	0.40 ± 0.08	0.27 ± 0.08	0.32 ± 0.04	0.30 ± 0.03	0.38 ± 0.07	0.37 ± 0.05	< 0.0001***
DO (mg/L)	9.8 ± 1.8	10.7 ± 1.5	10.2 ± 1.5	10.1 ± 1.7	9.92 ± 1.70	10.2 ± 1.6	9.08 ± 1.54	0.336
Conductivity (ms/m)	45.9 ± 8.5	50.1 ± 5.4	40.2 ± 8.5	49.0 ± 13.8	42.0 ± 7.0	50.5 ± 5.6	48.6 ± 7.7	< 0.05*
COD_Mn_ (mg/L)	3.6 ± 0.8	4.8 ± 0.7	3.8 ± 0.8	4.9 ± 1.0	3.6 ± 0.6	4.6 ± 0.7	4.0 ± 0.7	< 0.0001***
BOD_5_ (mg/L)	1.4 ± 0.5	3.0 ± 1.3	2.1 ± 1.4	3.4 ± 1.4	1.6 ± 0.3	2.6 ± 0.7	2.8 ± 0.7	< 0.0001***
TN (mg/L)	1.44 ± 0.68	1.97 ± 1.11	1.75 ± 1.07	2.67 ± 1.05	1.39 ± 0.73	1.69 ± 0.64	1.88 ± 0.74	< 0.05*
NH_4_^+^-N (mg/L)	0.07 ± 0.04	0.10 ± 0.05	0.103 ± 0.105	0.228 ± 0.229	0.08 ± 0.06	0.10 ± 0.06	0.21 ± 0.14	< 0.05*
TP (mg/L)	0.04 ± 0.02	0.07 ± 0.04	0.06 ± 0.03	0.10 ± 0.02	0.05 ± 0.02	0.06 ± 0.03	0.06 ± 0.02	< 0.0005***
Chl *a* (μg/L)	7.25 ± 4.58	69.2 ± 109	16.2 ± 2.4	64.1 ± 7.6	24.2 ± 44.6	14.8 ± 6.6	13.9 ± 7.7	< 0.0005***
COD_Cr_ (mg/L)	20 ± 5	25 ± 7	19 ± 5	25 ± 9	19 ± 6	25 ± 6	20 ± 6	0.065
F^−^ (mg/L)	0.621 ± 0.079	0.632 ± 0.076	0.615 ± 0.100	0.636 ± 0.104	0.607 ± 0.102	0.639 ± 0.065	0.553 ± 0.043	0.123
As (mg/L)	0.0017 ± 0.008	0.0029 ± 0.0024	0.0019 ± 0.0006	0.0026 ± 0.0019	0.0019 ± 0.004	0.0027 ± 0.0021	0.0023 ± 0.0014	0.760
Cu (mg/L)	0.0039 ± 0.0033	0.0049 ± 0.0055	0.0034 ± 0.0028	0.0044 ± 0.0037	0.0035 ± 0.0027	0.0048 ± 0.0057	0.0043 ± 0.0046	0.998
Pb (mg/L)	0.0043 ± 0.0058	0.0056 ± 0.0037	0.0028 ± 0.0033	0.0044 ± 0.0053	0.0042 ± 0.0061	0.0050 ± 0.0044	0.0073 ± 0.0066	0.425
MC-LR (μg/L)	0.069 ± 0.036	0.352 ± 0.349	0.091 ± 0.039	0.242 ± 0.196	0.098 ± 0.058	0.234 ± 0.281	0.116 ± 0.081	< 0.005**
NPR	55.7 ± 45.4	48.6 ± 63.0	32.1 ± 19.0	28.0 ± 13.4	33.1 ± 19.4	48.2 ± 62.9	35.2 ± 15.8	0.690
STSI	49.3 ± 3.3	57.6 ± 6.3	54.0 ± 4.7	61.0 ± 5.1	52.5 ± 4.0	54.5 ± 2.7	54.1 ± 2.5	< 0.00005***
Trophic state	Mesotrophication	Eutrophication	Eutrophication	Supereutrophication	Eutrophication	Eutrophication	Eutrophication	/

### Phytoplankton community composition and diversity

During a period of January to December of 2015, 124 taxa of phytoplankton, including eight phyla 21 taxa *Cyanophyta*, 63 *Chlorophyta*, 25 taxa of *Bacillariophyta*, 3 *Cryptophyta*, 4 *Pyrrophyta*, 7 *Euglenophyta*, and 1 *Chrysophyta*, were found in Tai Lake. These taxa belonged to 68 genera, including 13 genera of *Cyanophyta*, 27 genera of *Chlorophyta*, 17 genera of *Bacillariophyta*, 2 genera of *Cryptophyta*, 4 genera of *Pyrrophyta*, 4 genera of *Euglenophyta*, and 1 genera of *Chrysophyta*, were observed. Over the 12 months, relatively large densities of phytoplankton were observed at all seven locations in Tai Lake (Additional file 1: Fig. S2), with the greatest density of approximately 9.0 × 10^8^ cells/L at site TH4 in July. The second greatest density of phytoplankton was 7.4 × 10^8^ cells/L, which was observed at TH3 in July. Among all these monitored locations, TH7 in Gonghu Bay exhibited lesser densities of phytoplankton, ranging from 2.1 × 10^6^ to 6.8 × 10^7^ cells/L over the 12 months (*p *= 0.009).

**Fig. 2:**
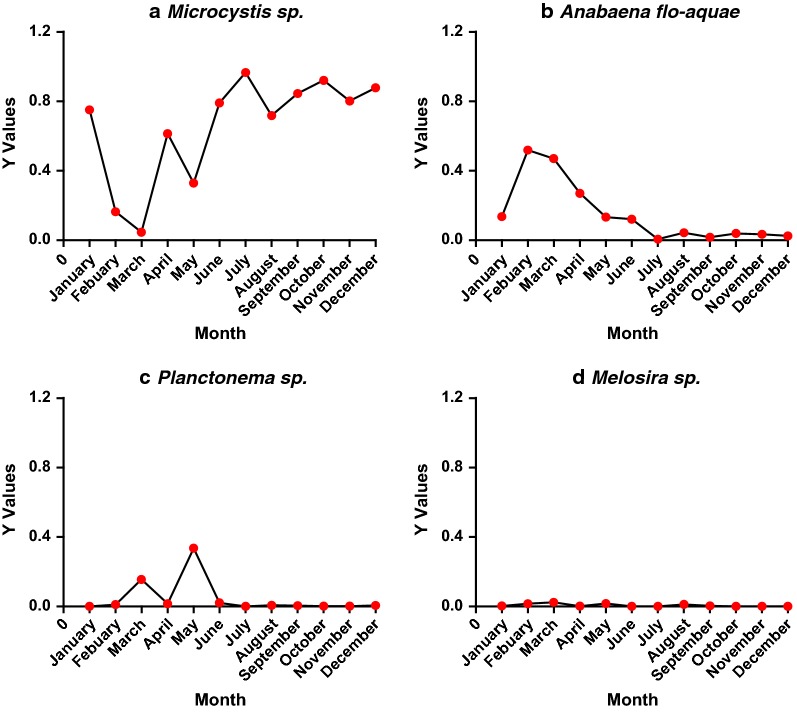
Percentages of dominant taxa (*Microcystis* sp., *Anabaena flos-aquae*, *Planctonema* sp. and *Melosira* sp.) in phytoplankton community and their temporal changes from January to December of 2015 in Tai Lake

In the present study, *Cyanophyta* was the dominant phylum in Tai Lake, with a mean percentage of abundance of 79.5%, followed by *Chlorophyta*, *Bacillariophyta* and *Cryptophyta*, with relative abundances of 12.9, 6.2 and 1.3%, respectively. Assemblages of phytoplankton in Tai Lake were dominated by *Cyanophyta,* including *Microcystis* sp. (*Y* = 0.80) and *Dolichospermum flos*-*aquae* (*Y* = 0.08). Based on monthly data, *Dolichospermum flos*-*aquae* was the dominant species in Tai Lake from February to March; *Planctonema* sp. of *Chlorophyta* was the most dominant species in May, while *Microcystis* sp. was dominant taxa in the nine other months (Fig. 2). In addition, *Microcystis* sp. was the absolutely dominant taxa from June to December. Meanwhile, average percentage of *Microcystis* sp. density in the phytoplankton community at TH4 was greatest to about 65.9%, followed by TH2 and TH6, with 63.0 and 57.0%, respectively (Table 2). Phytoplankton indicators at the seven locations in Tai Lake are shown in Table 2. The diversity of *H*′ and *J* at TH5 was greatest among the seven locations, while TH4 was the least (*p *< 0.01). Differences in phytoplankton indicators were significant among four seasons in Tai Lake. Diversity (*D*_s_, *H*′ and *D*_b_) and evenness (*J*) of phytoplankton communities in spring were greatest among four seasons in Tai Lake, while diversity of phytoplankton was least in summer (Additional file 1: Fig. S3). Differences in structures of phytoplankton communities were significant among seven locations in Tai Lake (MRPP: *A* = 0.031, *p *< 0.05), and differences in structures of communities of phytoplankton were also significant among months (MRPP: *A* = 0.242, *p *< 0.01).

**Table 2:** Percentages (mean ± SD) of densities of dominant taxa and values (mean ± SD) of phytoplankton indicators (diversity) at seven sampling sites from January to December of 2015 in Tai Lake

Items	TH1	TH2	TH3	TH4	TH5	TH6	TH7	*P*
P *Cyanophyta* %	76.2 ± 23.3	87.3 ± 19.7	86.8 ± 16.4	96.2 ± 3.5	69.5 ± 24.0	85.2 ± 20.0	55.4 ± 37.5	**
P *Chlorophyta* %	18.9 ± 21.6	6.1 ± 9.4	11.0 ± 16.0	2.3 ± 2.4	25.5 ± 21.4	9.2 ± 13.3	17.3 ± 25.5	*
P *Bacillariophyta* %	3.7 ± 3.1	5.7 ± 9.4	1.7 ± 1.6	1.2 ± 1.4	3.6 ± 3.4	4.7 ± 7.1	22.5 ± 28.8	*
P *Cryptophyta* %	1.1 ± 0.9	0.8 ± 1.6	0.4 ± 0.3	0.3 ± 0.2	1.3 ± 0.7	0.8 ± 1.3	4.5 ± 8.5	**
P *Microcystis* sp. %	50.3 ± 32.8	63.0 ± 35.3	45.1 ± 32.8	65.9 ± 36.5	40.7 ± 29.1	56.9 ± 34.7	44.0 ± 34.0	n.s.
P *Anabaena flos-aquae* %	18.2 ± 18.7	18.4 ± 21.0	26.6 ± 24.8	26.6 ± 33.0	24.3 ± 23.6	15.3 ± 20.7	1.8 ± 3.6	*
P *Planctonema* sp. %	18.9 ± 21.6	6.1 ± 9.4	11.0 ± 16.0	2.3 ± 2.4	25.5 ± 21.4	9.2 ± 13.3	17.3 ± 25.5	**
P *Melosira* sp. %	1.4 ± 2.2	1.6 ± 2.2	1.1 ± 1.3	0.6 ± 0.8	2.3 ± 2.8	0.6 ± 0.9	1.3 ± 2.9	n.s.
Taxa-*S*	14 ± 2	15 ± 6	19 ± 8	15 ± 4	15 ± 4	17 ± 5	14 ± 3	n.s.
*D*_s_	0.51 ± 0.25	0.39 ± 0.26	0.53 ± 0.24	0.27 ± 0.22	0.57 ± 0.16	0.46 ± 0.26	0.52 ± 0.21	n.s.
*H*′	1.14 ± 0.55	0.89 ± 0.58	1.21 ± 0.62	0.61 ± 0.46	1.28 ± 0.43	1.11 ± 0.69	1.24 ± 0.50	*
*J*	0.26 ± 0.12	0.20 ± 0.11	0.22 ± 0.11	0.14 ± 0.07	0.26 ± 0.09	0.21 ± 0.11	0.29 ± 0.13	*
*D*_b_	0.23 ± 0.16	0.17 ± 0.17	0.25 ± 0.17	0.10 ± 0.10	0.26 ± 0.15	0.24 ± 0.24	0.21 ± 0.14	n.s.

P *Cyanophyta*: percent of *Cyanophyta* density among the phytoplankton community; P *Chlorophyta*: percent of *Chlorophyta* density among the phytoplankton community; P *Bacillariophyta*: percent of *Bacillariophyta* density among the phytoplankton community; P *Cryptophyta*: percent of *Cryptophyta* density among the phytoplankton community; P *Microcystis* sp.: percent of *Microcystis* sp. density among the phytoplankton community

* *p *< 0.05; ** *p *< 0.005; *n.s* not significant

### Relationships between species composition and environmental variables

**Fig. 3:**
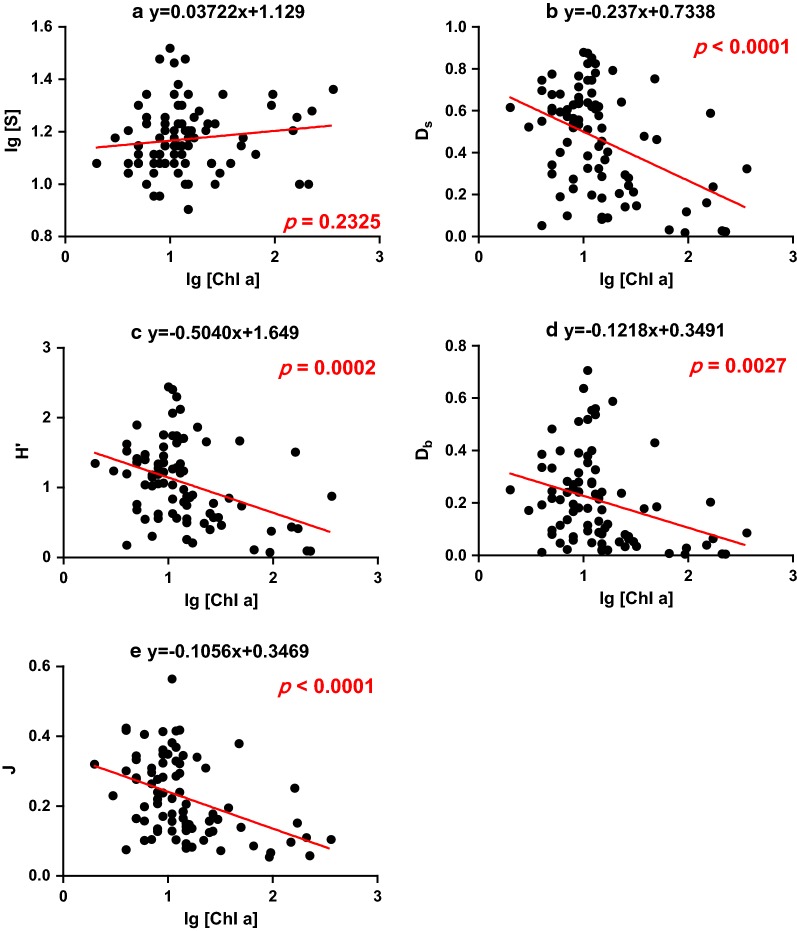
Scatter plots and linear regressions between productivity of phytoplankton (expressed in Chl *a*; log transformed) and measures of diversity of the phytoplankton community (*S* (log transformed), *D*_s_, *H*′, *D*_b_ and *J*) in Tai Lake. The curves were fitted based on *n* = 84 data points. The equation and *p* value for the specific linear regressions are given in each panel

Using a linear regression analysis, the relationships between phytoplankton productivity and diversity of *S*, *D*_s_, *H*′, *J*, and *D*_b_ are shown in Fig. 3 (*r*^2^ = 0.132, *p *= 0.232; R *r*^2^ = − 0.432, *p *< 0.001; *r*^2^ = − 0.392, *p *< 0.001; *r*^2^ = − 0.418, *p *< 0.001; *r*^2^ = − 0.323, *p *< 0.01, respectively). The species richness was positively, though not significantly, associated with productivity of phytoplankton (Fig. 3a), while significantly inverse relationships for the other four metrics of *D*_s_, *H*′, *J*, and *D*_b_ (Fig. 3b–e).

RDA was carried out on 17 environmental variables against Hellinger-transformed abundance species data with 999 permutations and significant relationships were determined by use of a global test (*F* = 2.446, *p *< 0.001). Among the 17 environmental variables, 11 parameters (WT, pH, SD, DO, COD_Mn_, BOD_5_, TP, COD_Cr_, F^−^, As and MC-LR) were significantly correlated (*p* < 0.05, 999 Monte Carlo permutations, Table 3) and accounted for 33.5% of the variance in the algae data. The first RDA axis, representing 21.5% variance of the species data, was significantly associated with WT, pH, SD, DO, COD_Cr_, F^−^, As and MC-LR. RDA axis 2 contributed additional 6.3% to the explained variance and was characterized significantly by COD_Mn_, BOD_5_ and TP (Table 3).

**Table 3:** Results of forward selection with 999 Monte Carlo permutations of environmental variables for species composition

Variables	RDA1	RDA2	*R* ^2^	*P* value
WT	*0.892*	0.452	0.240	0.001***
pH	*0.769*	0.639	0.110	0.013*
SD	*0.960*	− 0.279	0.086	0.017*
DO	− *0.738*	− 0.675	0.201	0.001***
Conductivity	− 0.999	0.053	0.063	0.080
COD_Mn_	− 0.036	− *0.999*	0.129	0.002**
BOD_5_	0.662	− *0.750*	0.100	0.016*
TN	− 0.967	− 0.256	0.023	0.36
NH_4_^+^–N	0.973	− 0.232	0.025	0.35
TP	0.603	− *0.798*	0.080	0.026*
COD_Cr_	− *0.943*	− 0.334	0.091	0.025*
F^−^	− *0.884*	− 0.467	0.417	0.001***
As	*0.982*	− 0.190	0.128	0.005**
Cu	− 0.859	0.511	0.007	0.764
Pb	0.678	0.735	0.063	0.069
MC-LR	*0.803*	− 0.596	0.108	0.007**
NPR	− 0.976	0.219	0.025	0.347

Correlations with RDA1 and RDA2, *R*^2^ and *p* values are shown. Significance was expressed as *p < 0.05, **p < 0.01, and ***p < 0.001

### Factors associating with phytoplankton indicators

Relationships among various indices, including diversity indices, dominant taxa and environmental variables were analyzed by use of generalized linear models (GLMs). WT, COD_Mn_, BOD_5_, MC-LR and NPR were the main factors that affected most parameters (Table 4). Several parameters, such as Chl *a*, *S*, total density, *Microcystis* sp., *Melosira* sp., *Cyanophyta*, *Chlorophyta*, and percent of *Dolichospermum flos-aquae* density were significantly related with WT (Table 4). Phytoplankton diversity indices (*H*′, *J* and *D*_b_) were significantly related with BOD_5_, MC-LR and NPR.

**Table 4:** Results of the best approximating generalized linear model (GLM), with Gaussian error distribution to explain the variation in phytoplankton indices (*n* = 84) and densities of dominant taxa

Chl *a*	Variables	Intercept	WT	DO	Conductivity	BOD_5_	TP	As				
	*t* value	− 3.279**	4.387***	3.349**	− 2.381*	4.59***	3.87***	− 3.379**				
	AIC	869.64										
*S*	Variables	Intercept	WT	pH	DO	BOD_5_	TP	As	NPR			
	*t* value	2.447*	2.829**	− 2.509*	1.841	1.508	− 1.827	1.471	1.787			
	AIC	495.25										
*D*_s_	Variables	Intercept	WT	DO	BOD_5_	TP	F^−^					
	*t* value	2.593*	− 1.543	− 1.848	− 1.375	− 2.654**	1.752					
	AIC	− 9.23										
*H*′	Variables	Intercept	BOD_5_	MC-LR	NPR							
	*t* value	9.066***	− 2.264*	− 2.482*	3.024**							
	AIC	136.04										
*J*	Variables	Intercept	BOD_5_	MC-LR	NPR							
	*t* value	10.15***	− 2.524*	− 2.439*	2.02*							
	AIC	− 135.33										
*D*_b_	Variables	Intercept	BOD_5_	MC-LR	NPR							
	*t* value	6.044***	− 1.966	− 2.401*	3.676***							
	AIC	− 71.62										
Total density	Variables	Intercept	WT	SD	DO	Conductivity	COD_Mn_	BOD_5_	NH_4_^+^− N	TP	As	MC-LR
	*t* value	− 2.304*	4.467***	− 1.875	4.078***	− 1.607	− 4.226***	8.436***	− 1.628	1.836	− 2.322*	1.868
	AIC	2169.30										
*Microcystis* sp.	Variables	Intercept	WT	SD	DO	Conductivity	COD_Mn_	BOD_5_	NH_4_^+^− N	TP	As	MC-LR
	*t* value	− 2.246*	4.366***	− 1.828	3.938***	− 1.722	− 4.133***	8.402***	− 1.786	1.825	− 2.24*	1.854
	AIC	2163.82										
*Anabaena flos*-*aquae*	Variables	Intercept	COD_Mn_	BOD_5_	TP	F^−^						
	*t* value	− 2.65**	− 2.662**	2.514*	2.152*	3.893***						
	AIC	1860.70										
*Planctonema* sp.	Variables	Intercept	WT	BOD_5_	TN	As						
	*t* value	1.045	1.955	− 2.176*	1.42	− 1.523						
	AIC	1624.20										
*Melosira* sp.	Variables	Intercept	WT	pH	DO	TN	TP					
	*t* value	− 0.195	3.929***	− 1.533	3.394**	2.375*	− 2.529*					
	AIC	1245.40										
*Cyanophyta*	Variables	Intercept	WT	SD	DO	Conductivity	COD_Mn_	BOD_5_	NH_4_^+^–N	TP	As	MC-LR
	*t* value	− 2.288*	4.357***	− 1.794	4.019***	− 1.654	− 4.185***	8.422***	− 1.662	1.874	− 2.233*	1.829
	AIC	2170.00										
*Chlorophyta*	Variables	Intercept	WT	BOD_5_	As							
	*t* value	1.969	2.468*	− 1.444	− 2.189*							
	AIC	1626.10										
*Bacillariophyta*	Variables	Intercept	TN									
	*t* value	0.748	1.627									
	AIC	1503.20										
P.*Microcystis* sp.	Variables	Intercept	WT	BOD_5_	TN	COD_Cr_	F^−^	Pb	MC-LR			
	*t* value	3.071**	1.976	3.08**	− 2.409*	− 1.926	− 1.63	− 1.483	2.538*			
	AIC	807.26										
P.*Anabaena flos-aquae*	Variables	Intercept	WT	SD	COD_Cr_	F						
	*t* value	− 1.77	− 3.129**	− 2.739**	2.466*	4.945***						
	AIC	712.85										

Selected variables, *t* values and AIC are given. Significance was expressed as *p *< 0.1, * p < 0.05, ** p < 0.01, and *** p < 0.001. The best approximating model was selected by Akaike’s information criterion (AIC)

## Discussion

### Temporal changes in phytoplankton communities of Tai Lake

Tai Lake is a large shallow lake, which provides hospitable environment for phytoplankton, and phytoplankton communities have changed over time (Table 5). In the 1960s, there were 91 genera of phytoplankton belonging to eight phyla, which were more frequently observed compared to the present study. Although the mean percent of *Cyanophyta* among constituents of phytoplankton density was 96.6% in the western area of the lake in 1960s, the greatest density of total phytoplankton was only 6.6 × 10^5^ cells/L in Tai Lake [61], and were much less than densities observed in this study (Additional file 1: Fig. S2). Meanwhile, at that time, the community was dominated by *Bacillariophyta*–*Chlorophyta* in TH3, which is in the southwest of the lake. Concentrations of nutrients (Nitrogen: 0.15 mg/L, PO_4_^3−^P: 0.05 mg/L) and pollutants (COD_Mn_: 1.90 mg/L) in the whole Tai Lake were also less than those measured here (Table 1). Since 1980s, cell density of phytoplankton increased in the rate of 5.8 times per year. Subsequently, blooms of cyanobacteria led to a drinking water crisis in summer of 2007; numbers of phytoplankton in Meiliang Bay ranged from 3.0 × 10^6^ to 3.7 × 10^9^ cells/L, with a maximum density of phytoplankton occurring in June in 2008 [62].

**Table 5:** Comparaison of changes in numbers of phytoplankton communities at the genus level of Tai Lake in 1960s and the present study (2015)

The studies	*Cyanophyta*	*Chlorophyta*	*Bacillariophyta*	*Cryptophyta*	*Pyrrophyta*	*Euglenophyta*	*Chrysophyta*	*Xanthophyceae*	Total number of genus
1960s	15	48	18	0	2	5	1	2	91
Our study (2015)	13	27	17	2	4	4	1	0	68

The structure of the phytoplankton community in Tai Lake is still changing but in a good direction. In the present study, *Microcystis* sp. and *Dolichospermum flos*-*aquae* of *Cyanophyta*, and *Planctonema* sp. of *Chlorophyta* were still predominant taxa observed (Fig. 2). Numbers of phytoplankton at the seven locations studied ranged from 2.5 × 10^6^ to 9.0 × 10^8^ cells/L. In Meiliang Bay, the number of phytoplankton ranged from 3.2 × 10^6^ to 2.4 × 10^8^ cells/L, that was less than that in 2008 [62]. This result was probably because nitrogen and phosphorus have been controlled in Tai Lake since the drinking water crisis of 2007. Many ecological restoration projects used a wetland system to treat the rural wastewater removing nitrogen in the Lake [63]. Therefore, concentrations of TN and TP observed in 2015 were less than reported before [64].

In the present study, numbers of cells of cyanobacteria were still high in the lake, and concentrations of N and P were still greater than the thresholds of 0.80 and 0.05 mg/L, respectively, limiting blooms of *Microcystis* in Tai Lake [38]. In 2015, 68 genera of phytoplankton among seven phyla were observed, which was less than the 91 genera in eight phyla in 1960s [61]. The dominance (Y) of *Microcystis* sp. in Tai Lake was the greatest (Fig. 2), which was consistent with results of a previous study that phytoplankton of eutrophic shallow lakes was frequently dominated by one species or species of the same functional group, resulting in species-pure algal assemblages [65]. Phyla including *Pyrrophyta*, *Euglenophyta* and *Chrysophyta* appeared rarely in Tai Lake, which is consistent with the previous studies in eutrophic waters [66]. As nutrient concentrations and phytoplankton blooms were increasing, transparency was low in Tai Lake and cyanobacteria can outcompetes subsurface phytoplankton species by reducing photosynthetically available light through shading [67, 68].

### Relationships between phytoplankton productivity and diversity

In general, diversity curves are either concave-down or exhibit increasing functions with productivity [55, 69]. Retention and concentrations of nutrients are relatively high and residence time is long in Tai Lake [35]. Thus, the morphometric and hydrologic parameters are favorable for development of great biomasses of phytoplankton. In the present study, various shapes of relationships between productivity and diversity were observed. Diversity metrics of *H′*, *D*_s_, *D*_b_ and *J* showed similar changes along the productivity gradient in Tai Lake (Fig. 3b–e). An inverse relationship was observed between diversity of the phytoplankton community and primary production in Tai Lake. Results of a previous study [65] have demonstrated that dominance of bloom-forming cyanobacteria that can more successfully compete for light, exerted strong negative effects on other species of phytoplankton. In contrast, the logarithm of species richness was slightly and positively correlated with primary productivity in Tai Lake, which was consistent with previous findings [70], showing that productivity could determine the upper values of diversity metrics and lesser values of species richness were found in the whole productivity range. The results of previous studies that focused on the decisive role of physical disturbances in determining diversity and the productivity–diversity relationship for phytoplankton in lakes, suggested that the relationships between productivity and diversity can be used to assess ecological state [70, 71]. The results of this study indicated that diversity of phytoplankton communities is significantly related to trophic status and overall primary production in Tai Lake.

### Factors effecting on structure of the phytoplankton community

WT, pH, COD_Mn_, BOD_5_, TP, NPR, As and MC-LR were the main factors effecting structures of the phytoplankton community in Tai Lake (Tables 3 and 4). The pH (from 7.29 to 9.07) in 2015 was greater than those (from 7.30 to 8.10) observed in the 1960s, and numbers of cyanobacteria was greater than they were in 1960s [61]. When photosynthesis of algae consumed inorganic carbon, the carbonate-buffering capacity of the Tai lake was decreased, which increased pH [72, 73]. The researchers explained that photosynthesis is increasing pH by CO_2_ consumption; cyanobacteria have the ability to use HCO_3_^−^ [74] which could be part of their competitive advantage under higher pH conditions.

Temperature is a crucial factor affecting the composition of the phytoplankton community, and greater numbers of cells usually observe in warmer seasons [14]. Furthermore, temperature is the most important factor controlling which phytoplankton taxa is present in freshwater lakes [75, 76]. Results of the current study are consistent with results of previous studies [77–80], and showing that cyanobacteria-dominated phytoplankton communities in warmer seasons. Once critical concentrations of nutrients were exceeded, temperature was the principal factor driving blooms of *Microcystis* [81, 82]. Compared with *Bacillariophyta*, *Cyanophyta* have a competitive advantage under conditions of greater temperature, especially when temperature of water exceeds 25 °C [13, 83]. In addition, Gala and Giesy [84] demonstrated that UV light can penetrate into water, especially in shallow lakes, and that phytoplankton exhibited differential sensitivities. Hence, there can be seasonal changes in relative densities due to these differential sensitivities and cyanobacteria are the least sensitive class of taxa. Even though arsenic (As) can isomorphically substitute for phosphorus (P), it was not conducive to growth of algae. In the present study, significantly negative correlations between concentrations of As and concentrations of Chl *a* and total numbers of phytoplankton were observed (Table 4). Phytoplankton can be involved in geochemical cycling of As in aquatic ecosystems [85, 86]. In fact, there are some phytoplankton, especially cyanobacteria, that are resistant to adverse effects of As [87, 88], because they contain a class of proteins similar to metallothioneins [89] and could accumulate and transform As. Concentrations of phosphorus cannot rescue other species from the toxic effects of As.

Since growth and reproduction of phytoplankton requires absorption of nutrients, such as N and P, concentrations of these nutrients have an important impact on structures of phytoplankton communities [38, 90, 91]. Species richness of phytoplankton changed as a function of the ratio of N to P (NPR) [92, 93]. In Tai Lake, *Microcystis* spp. was the most competitive among taxa of phytoplankton. This observation is supported by the significantly negative relationship between concentrations of MC-LR and indicators of diversity (*H*′, *J* and *D*_b_) of the phytoplankton community (Table 4). Results of this study also demonstrated that *Cyanophyta*, such as *Microcystis* spp., had a negative effect on growth and relative proportions of other phytoplankton. Toxins derived from cyanobacteria can reduce the growth of diatoms [94]. Specifically, *Microcystis* spp. and *Pseudanabaena* spp. can produce extracellular products that are toxic to both aquatic organisms and humans [95–97]. Our findings indicated that blooms of *Microcystis* resulted in less stability of structure of the phytoplankton community. This result was consistent with a previous study, in which extracellular products of *Cyanophyta* promoted clustering of cyanobacterial cells, which might be due to the toxins inducing algal cells to release polysaccharides [98]. Furthermore, formation of clusters helped cyanobacteria in escaping predation by zooplankton [99]. Collectively, results of the present study demonstrated that MC-LR was significantly and negatively associated with diversity of aquatic communities, which were consistent with results of a previous study (Li et al. [32]).

## Conclusions

The present study investigated the structure of the phytoplankton community over a period of January to December of 2015 in Tai Lake and demonstrated that the structure of phytoplankton community was changed as the changing concentrations of nutrients and other stressors. WT, pH, permanganate index (COD_Mn_), BOD_5_, TP, As, TN/TP ratio (NPR) and MC-LR were the main factors that influenced the structure of the phytoplankton community in Tai Lake. The results demonstrated that Simpson, Shannon–Wiener, Berger and Parker and the Pielou evenness indices could be used to assess and monitor for status and trends in water quality of Tai Lake. This research will be helpful in understanding the changing environment on biodiversity in aquatic ecosystems.

## Additional file

**Additional file 1: Figure S1.** Profiles of diversity represented by one-parametric Renyi diversity index for two hypothetical assemblages, denoted by A and B. Vertical dotted lines denote values of the scale parameter (measured along the *x*-axis), which provides classical diversity index statistics, such as number of species, Shannon, Simpson, and Berger–Parker index of diversity. **Figure S2.** Densities of phytoplankton taxa of seven sampling sites in Tai Lake. **Figure S3.** Diversities of phytoplankton communities at four different seasons in Tai Lake. **Table S1.** Synthesized index of trophic state (STSI), trophic state, and qualitative descriptor of water quality. **Table S2.** Pairwise Spearman’s correlations coefficients (*ρ*) among environmental variables.
