# Supplementary material for: Factors associated with blooms of cyanobacteria in a large shallow lake, China
Source: Environ Sci Eur. 2018 Jul 28;30(1):27. doi: 10.1186/s12302-018-0152-2 (PMC6096964; doi:10.1186/s12302-018-0152-2)
Supplement: Supplementary file 1 — Additional file 1: Figure S1. Profiles of diversity represented by one-parametric Renyi diversity index for two hypothetical assemblages, denoted by A and B. Vertical dotted lines denote values of the scale parameter (measured along the x-axis), which provides classical diversity index statistics, such as number of species, Shannon, Simpson, and Berger–Parker index of diversity. Figure S2. Densities of phytoplankton taxa of seven sampling sites in Tai Lake. Figure S3. Diversities of phytoplankton communities at four different seasons in Tai Lake. Table S1. Synthesized index of trophic state (STSI), trophic state, and qualitative descriptor of water quality. Table S2. Pairwise Spearman’s correlations coefficients (ρ) among environmental variables. [file 12302_2018_152_MOESM1_ESM.docx]

**Factors Associated with Blooms of Cyanobacteria in a large shallow lake, China**

Di Li E-mails: [ld@jshb.gov.cn](mailto:ld@jshb.gov.cn); Naicheng Wu E-mails: [nwu@hydrology.uni-kiel.de](mailto:nwu@hydrology.uni-kiel.de); Song Tang E-mails: [tangsong@nieh.chinacdc.cn](mailto:tangsong@nieh.chinacdc.cn); Guanyong Su E-mails: [sugy@njust.edu.cn](mailto:sugy@njust.edu.cn); Xuwen Li E-mails: [lxw241@163.com](mailto:lxw241@163.com); Yong Zhang E-mails: [zhangyong@jshb.gov.cn](mailto:zhangyong@jshb.gov.cn); Guoxiang Wang E-mails: [442371719@qq.com](mailto:442371719@qq.com); Junyi Zhang E-mails: [blocksharon@163.com](mailto:blocksharon@163.com); Hongling Liu E-mails: [hlliu@nju.edu.cn](mailto:hlliu@nju.edu.cn); Markus Hecker E-mails: [markus.hecker@usask.ca](mailto:markus.hecker@usask.ca); John P. Giesy E-mails: [JGiesy@aol.com](mailto:JGiesy@aol.com); Hongxia Yu E-mails: [yuhx@nju.edu.cn](mailto:yuhx@nju.edu.cn)

^1^ State Key Laboratory of Pollution Control and Resource Reuse, School of the Environment, Nanjing University, Nanjing, Jiangsu 210046, China

^2^ Jiangsu Environmental Monitoring Center, Nanjing, Jiangsu 210036, China

^3^ Department of Hydrology and Water Resources Management, Kiel University, Kiel 24118, Germany

^4^ National Institute of Environmental Health, Chinese Center for Disease Control and Prevention, Beijing 100021, China

^5^ Jiangsu Key Laboratory of Chemical Pollution Control and Resources Reuse, School of Environmental and Biological Engineering, Nanjing University of Science and Technology, Nanjing 210094, China

^6^ School of the Environment, Nanjing Normal University, Nanjing, Jiangsu 210023, China

^7^ Wuxi Environmental Monitoring Center, Wuxi, Jiangsu 214000, China

^8^ Toxicology Centre, University of Saskatchewan, Saskatoon, SK S7N 5B3, Canada.

^9^ School of Environment and Sustainability, University of Saskatchewan, Saskatoon, SK S7N 5C3, Canada.

^10^ Department of Veterinary Biomedical Sciences and Toxicology Centre, University of Saskatchewan, Saskatoon, Saskatchewan S7N5B3, Canada

^11^ Department of Zoology and Center for Integrative Toxicology, Michigan State University, East Lansing, Michigan 48824, United States

^12^ School of Biological Sciences, University of Hong Kong, Hong Kong, SAR China

^*^Corresponding author: Dr. Hongxia Yu, State Key Laboratory of Pollution Control and Resource Reuse, School of the Environment, Nanjing University, Nanjing, Jiangsu 210046, China

Tel: +86-25-89680356; Fax: +86-25-89680356; E-mails: [yuhx@nju.edu.cn](mailto:yuhx@nju.edu.cn)

**Figure S1.** Profiles of diversity represented by one-parametric Renyi diversity index for two hypothetical assemblages, denoted by A and B. Vertical dotted lines denote values of the scale parameter (measured along the x-axis), which provides classical diversity index statistics, such as number of species, Shannon, Simpson, and Berger-Parker index of diversity.


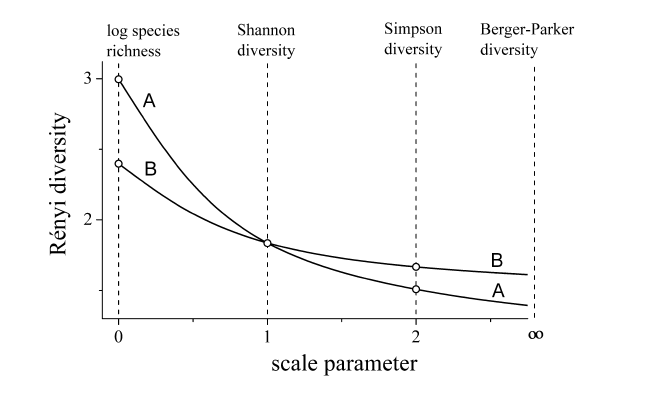


**Figure S2.** Densities of phytoplankton taxa of seven sampling sites in Tai Lake.


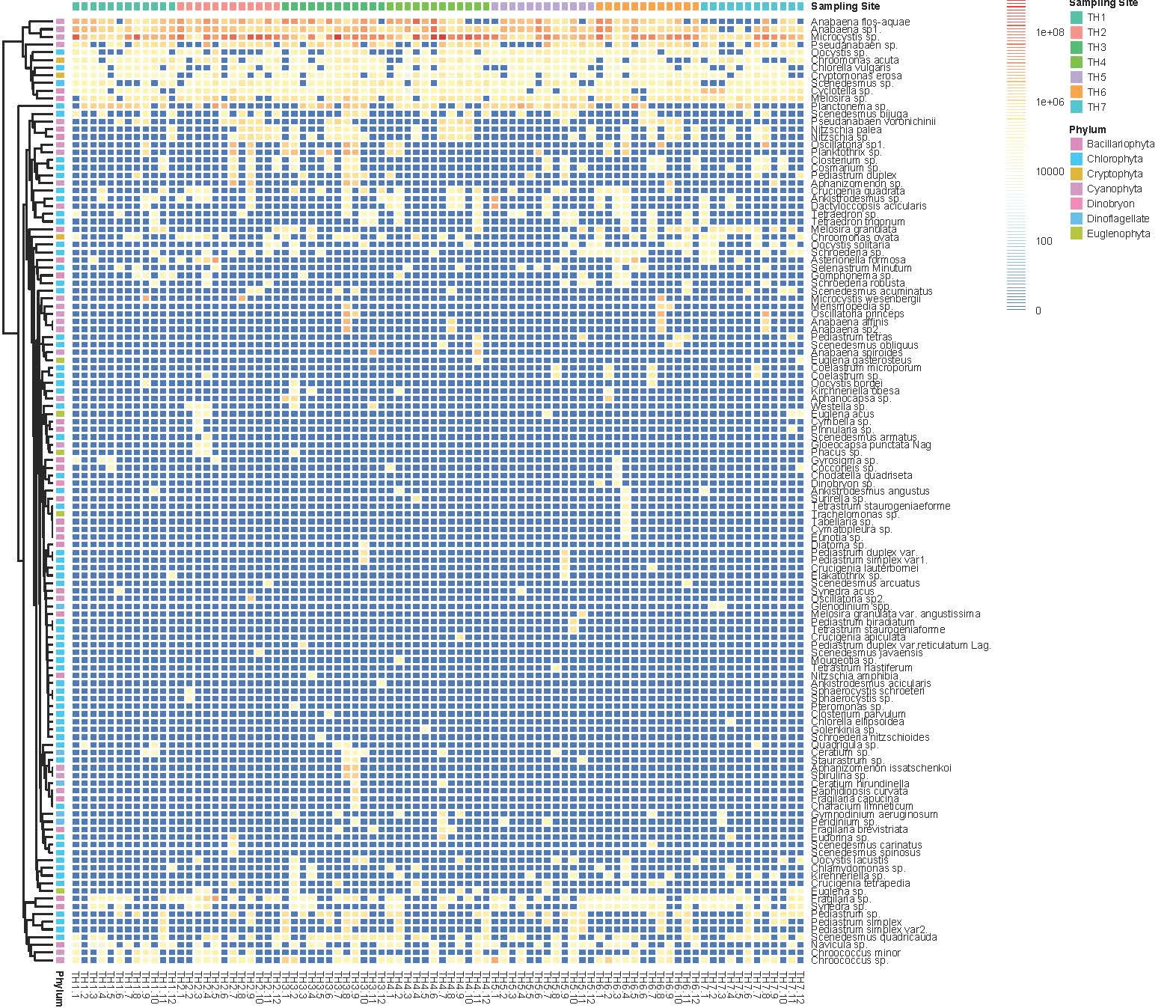


**Figure S3.** Diversities of phytoplankton communities at four different seasons in Tai Lake.

**Table S1.** Synthesized index of trophic state (STSI), trophic state, and qualitative descriptor of water quality.

| **Synthesized trophic state index (STSI)** | **Trophic state** | **Water quality** |
| --- | --- | --- |
| 0＜STSI≤30 | Oligotrophication | Excellent |
| 30＜STSI≤50 | Mesotrophication | Good |
| 50＜STSI≤60 | Eutrophication | Polluted |
| 60＜STSI≤70 | Supereutrophication | Superpolluted |
| 70＜STSI≤100 | Hypereutrophication | Hyperpolluted |

**Table S2.** Pairwise Spearman’s correlations coefficients (*ρ*) among environmental variables.

|  | WT | pH | SD | DO | Conductivity | CODMn | BOD5 | TN | NH4N | TP | CODCr | F | As | Cu | Pb | MCLR | NPR | STSI |
| --- | --- | --- | --- | --- | --- | --- | --- | --- | --- | --- | --- | --- | --- | --- | --- | --- | --- | --- |
| WT | 1.000 | 0.693 | 0.103 | -0.803 | 0.017 | -0.072 | 0.065 | -0.017 | 0.083 | 0.072 | -0.070 | -0.474 | 0.475 | 0.219 | 0.578 | 0.012 | -0.037 | 0.073 |
| pH | 0.693 | 1.000 | 0.238 | -0.449 | 0.266 | -0.061 | 0.019 | 0.002 | -0.117 | -0.039 | 0.042 | -0.236 | 0.306 | 0.152 | 0.459 | 0.191 | 0.094 | 0.021 |
| SD | 0.103 | 0.238 | 1.000 | -0.105 | 0.318 | 0.163 | 0.321 | 0.052 | 0.077 | -0.176 | 0.258 | -0.090 | 0.139 | -0.022 | 0.151 | 0.174 | 0.216 | -0.181 |
| DO | -0.803 | -0.449 | -0.105 | 1.000 | -0.093 | 0.277 | 0.030 | -0.036 | -0.179 | 0.015 | 0.115 | 0.432 | -0.343 | -0.336 | -0.512 | 0.174 | -0.043 | 0.100 |
| Conductivity | 0.017 | 0.266 | 0.318 | -0.093 | 1.000 | 0.081 | 0.238 | 0.360 | -0.061 | -0.041 | 0.506 | 0.215 | -0.273 | 0.022 | 0.169 | 0.016 | 0.329 | -0.013 |
| CODMn | -0.072 | -0.061 | 0.163 | 0.277 | 0.081 | 1.000 | 0.565 | 0.031 | 0.224 | 0.423 | 0.248 | 0.300 | 0.144 | -0.055 | 0.111 | 0.531 | -0.336 | 0.558 |
| BOD5 | 0.065 | 0.019 | 0.321 | 0.030 | 0.238 | 0.565 | 1.000 | 0.490 | 0.324 | 0.377 | 0.447 | 0.078 | 0.137 | 0.023 | 0.163 | 0.321 | 0.020 | 0.613 |
| TN | -0.017 | 0.002 | 0.052 | -0.036 | 0.360 | 0.031 | 0.490 | 1.000 | 0.200 | 0.237 | 0.464 | 0.080 | -0.199 | 0.057 | -0.079 | 0.064 | 0.551 | 0.415 |
| NH4N | 0.083 | -0.117 | 0.077 | -0.179 | -0.061 | 0.224 | 0.324 | 0.200 | 1.000 | 0.174 | -0.138 | -0.043 | 0.093 | 0.069 | 0.224 | -0.017 | -0.049 | 0.265 |
| TP | 0.072 | -0.039 | -0.176 | 0.015 | -0.041 | 0.423 | 0.377 | 0.237 | 0.174 | 1.000 | 0.040 | -0.133 | 0.278 | 0.047 | -0.079 | 0.398 | -0.653 | **0.818** |
| CODCr | -0.070 | 0.042 | 0.258 | 0.115 | 0.506 | 0.248 | 0.447 | 0.464 | -0.138 | 0.040 | 1.000 | 0.333 | -0.076 | -0.002 | -0.027 | 0.022 | 0.312 | 0.173 |
| F | -0.474 | -0.236 | -0.090 | 0.432 | 0.215 | 0.300 | 0.078 | 0.080 | -0.043 | -0.133 | 0.333 | 1.000 | -0.232 | -0.142 | -0.058 | 0.058 | 0.144 | -0.017 |
| As | 0.475 | 0.306 | 0.139 | -0.343 | -0.273 | 0.144 | 0.137 | -0.199 | 0.093 | 0.278 | -0.076 | -0.232 | 1.000 | -0.065 | 0.240 | 0.183 | -0.398 | 0.163 |
| Cu | 0.219 | 0.152 | -0.022 | -0.336 | 0.022 | -0.055 | 0.023 | 0.057 | 0.069 | 0.047 | -0.002 | -0.142 | -0.065 | 1.000 | 0.499 | -0.065 | 0.002 | 0.025 |
| Pb | 0.578 | 0.459 | 0.151 | -0.512 | 0.169 | 0.111 | 0.163 | -0.079 | 0.224 | -0.079 | -0.027 | -0.058 | 0.240 | 0.499 | 1.000 | 0.144 | 0.027 | -0.039 |
| MCLR | 0.012 | 0.191 | 0.174 | 0.174 | 0.016 | 0.531 | 0.321 | 0.064 | -0.017 | 0.398 | 0.022 | 0.058 | 0.183 | -0.065 | 0.144 | 1.000 | -0.284 | 0.391 |
| NPR | -0.037 | 0.094 | 0.216 | -0.043 | 0.329 | -0.336 | 0.020 | 0.551 | -0.049 | -0.653 | 0.312 | 0.144 | -0.398 | 0.002 | 0.027 | -0.284 | 1.000 | -0.372 |
| STSI | 0.073 | 0.021 | -0.181 | 0.100 | -0.013 | 0.558 | 0.613 | 0.415 | 0.265 | **0.818** | 0.173 | -0.017 | 0.163 | 0.025 | -0.039 | 0.391 | -0.372 | 1.000 |

Note: STSI was excluded due to its higher correlations with TP (bold numbers).
